# Individual and environmental correlates of objectively measured sedentary time in Dutch and Belgian adults

**DOI:** 10.1371/journal.pone.0186538

**Published:** 2017-10-17

**Authors:** Femke van Nassau, Joreintje D. Mackenbach, Sofie Compernolle, Ilse de Bourdeaudhuij, Jeroen Lakerveld, Hidde P. van der Ploeg

**Affiliations:** 1 Department of Public and Occupational Health, Amsterdam Public Health Research Institute, VU University Medical Center, Amsterdam, The Netherlands; 2 Department of Epidemiology and Biostatistics, Amsterdam Public Health Research Institute, VU University Medical Center, Amsterdam, The Netherlands; 3 Department of Movement and Sport Sciences, Ghent University, Ghent, Belgium; Medical University of Vienna, AUSTRIA

## Abstract

As the detrimental health effects of sedentary behaviour are well established, insight into the individual and environmental factors that influence adults’ sedentary behaviour is needed. Most studies to date rely on self-reported measures of sedentary time. Therefore, the aim of the current study was to examine individual and environmental correlates of objectively measured sedentary time in Dutch and Belgian adults. Between March and August 2014, Belgian (n = 133) and Dutch (n = 223) adults, recruited as sub-sample of the SPOTLIGHT survey, wore an ActiGraph accelerometer to provide objectively measured sedentary and moderate to vigorous physical activity time. Participants completed a questionnaire assessing sociodemographic (country of residence, age, gender and educational level), lifestyle (sleep, smoking, sugar-containing beverage consumption, alcohol intake), health (body mass index, self-rated health), work (employment status and type of work), happiness, physical environmental (owning a car, number of screens, socioeconomic status and residential density) and social environmental factors (social network, social cohesion). Univariate and multivariable regression analyses showed that Belgian participants had a lower odds of being sedentary for at least 9 hours per day compared to Dutch participants. Women, older participants and those meeting the WHO recommendation for physical activity were also less likely to sit for 9 hours or more per day. Participants doing (heavy) manual work or being in education, homemaker, unemployed had lower odds of being sedentary for at least 9 hours per day compared to participants with a sitting job. Those with a higher self-reported social network also had lower odds for sedentary time. No associations between physical and other social environmental characteristics and sedentary time were found. Our findings add to the growing evidence of factors associated with prolonged sedentary time in adults. These findings may be used to inform the development of strategies and interventions aimed at reducing sedentary time, and to identify high risk groups.

## Introduction

Evidence has been accumulating that prolonged sedentary behaviour is associated with an elevated risk of all-cause, cancer- and cardiovascular mortality [1;2]. Sedentary behaviour (i.e. any waking activity characterised by an energy expenditure ≤ 1.5 metabolic equivalents while in a sitting, reclining or lying posture[3]) comprises a major part of modern lifestyles: at work, during leisure time (watching television, using computers and handhelds) and during transportation.

In order to combat sedentary lifestyles through approaches that target high-risk groups, information on factors that are associated with (prolonged) sedentary behaviour is essential. To date, few consistent factors have been identified. A review by Rhodes et al. (2012) concluded that several sociodemographic factors (such as age and gender), and health and lifestyle related factors (such as physical activity (PA) and body mass index (BMI)), were linked to sedentary behaviour. They noted a lack of studies focusing on a relation with cognitive characteristics or ‘upstream’ characteristics in the social and physical environment [4].

A recent review took a more socio-ecological approach [5] and identified several individual level factors (i.e. being older, female, low levels of PA, high BMI, smoking and high calorie snack intake and frequent cell phone use were associated with higher sedentariness). Their review showed mixed results for interpersonal factors, such as marital status, number of children, social cohesion and social norms, as well as for environmental factors, such as indoor environment and characteristics in the neighbourhood environment. Lack of comparability across studies with regard to the associations under study as well as measurement instruments hindered drawing conclusions on associations with sedentary behaviour.

In addition, the review of Koohsari et al. specifically looked at neighbourhood environmental correlated of sedentary behaviour [6]. Living in an urban area with a variety of destinations nearby was associated with less sedentary behaviour during leisure time and transport. Other neighbourhood environmental attributes were not closely related to sedentary behaviour.

All three reviews reported a large variety of measurement tools/techniques of sedentary behaviour, ranging from TV viewing to socializing and sitting, mostly using self-reported measures of sedentary behaviour with often unknown validity [4–6]. Self-reported sedentary behaviour is susceptible to recall bias and social desirable answers, and it has been recommended to integrate more objective measures of sedentary behaviour in future studies. Therefore, the objective of the current study was to explore sociodemographic, lifestyle, health, work, psychological, physical home and neighbourhood and social neighbourhood correlates of objectively measured sedentary time in adults who participated in the SPOTLIGHT (‘Sustainable prevention of obesity through integrated strategies’) project in Belgium and the Netherlands. This is one of the first studies to explore associations between objective measures of sedentary time and both individual and environmental factors.

## Methods

A total of 6037 participants from 60 neighbourhoods in five countries participated in an online survey as part of the European SPOTLIGHT survey [7;8]. Data used for this study were from Belgian and Dutch participants only, as these were the only SPOTLIGHT countries that collected accelerometer data. The study was approved by the VU University Medical Center ethics committee (2012/314) and the Ghent University Hospital ethical committee (EC/2013/518) and all participants provided written informed consent.

### Participants

Sampling of neighbourhoods and recruitment of participants has been described in detail elsewhere [8]. Briefly, neighbourhood sampling was based on a combination of residential density and socioeconomic status (SES) data at neighbourhood level. In 12 randomly selected neighbourhoods in each country, a random sample of residential addresses was drawn from postal companies (the Netherlands), or public administration services (Belgium). Between February and September 2014, 55893 adults were recruited via postal invitation to participate in the online survey, of whom 6037 (10.8%) responded.

Between March and October 2014, 379 of the 1609 Dutch and 167 of the 1849 Belgian participants who completed the survey and left their phone number or email address to be contacted for potential future studies, were asked to participate in the accelerometer study. In total, 225 Dutch and 149 Belgian participants provided accelerometer data. Timing between the questionnaire and accelerometer assessment varied between participants, but was less than six months in all cases.

In the Netherlands, an accelerometer was sent to the participants’ home address including a written instruction on how to wear the device. In Belgium, researchers visited the participants to attach the accelerometer and explain procedures. Participants were asked to wear the device for seven consecutive days during waking hours. They were told to temporarily remove the device during water-based activities and to refit the device as soon as possible afterwards. Participants were asked to keep a monitoring log.

### Measures

#### Objectively measured sedentary behaviour

Participants were asked to wear the commonly used tri-axial accelerometer (ActiGraph GT3X+) fixed with an elastic belt on the right hip during waking hours. The GT3X+ activity monitor (ActiGraph, LLC, Fort Walton Beach, FL) is a commonly used small (51 x 41 x 15 mm), lightweight (27 grams) accelerometer. The ActiGraph provides activity counts, which can be converted into time spent in sedentary (<100 counts/min), light (100–2019 counts/min), moderate (2020–5998 counts/min), and vigorous intensity activity (>5999 counts/min) using established cut points for adults [9]. Non-wear time was defined as 60 minutes of consecutive zeroes, allowing for two interruptions of <100 counts per minute. Participants had to wear the accelerometer for at least 10 hr/d for it to be considered a valid day and only participants with four valid days were included in the analyses.

A recent meta-analysis suggests that the risk of all-cause mortality increases if adults sit more than around seven to eight self-reported hours per day [1]. The single item questionnaires generally used in the prospective cohort studies included in this meta-analysis tend to underestimate their self-reported daily time spent sitting [10;11]. As no standardized threshold for objective sedentary time is available, we dichotomized time spent sedentary in sitting 9 hours or less and more than 9 hours per day, which was close to the median sedentary time of 9.1 hr/d in the current study sample. And we also ran a sensitivity analysis for sitting more than 10 hours per day.

#### Individual and environmental correlates

We only included variables from the full SPOTLIGHT questionnaire that had a theoretical basis for possible associations with sedentary behaviour. (see copy of SPOTLIGHT questionnaire items used in this study in S1–S3 Questionnaires).

#### Sociodemographic factors

Information was obtained through the online survey on country of residence, age, gender and educational level. Age was recoded into 4 groups (i.e. <35 yr, 35–49 yr, 50–64yr and ≥65 yr). The item on educational level divided into low (no education/ completed primary school/ lower vocational education/ general secondary education), medium (secondary vocational or higher general secondary education), or high education (bachelor-education degree or higher) as defined in the ISCED.

#### Lifestyle factors

Moderate to vigorous physical activity (MVPA) was calculated by summing up moderate and vigorous intensity activity objectively measured by the accelerometer. We dichotomized MVPA into less than 150 minutes per week or 150 minutes or more, in line with the World Health Organization (WHO) physical activity recommendations for adults [12].

Sleep was assessed by the question: *‘How many hours per night do you sleep on average*?*’* The response options ranged from 4 to 16 h per night (in half-hourly intervals). Because both short and long sleep have previously been associated with unfavourable health outcomes [13;14], we classified sleep in two variables: normal sleep (7–9 hours per day) and short or long sleeper (<7 or >9 hours sleep per day) according to cut-off points used in previous studies [15].

Participants also indicated if they were a smoker, former smoker or non- smoker. Participants also reported the frequency of consuming sugar-containing beverages and alcoholic beverages both by a single item: *‘How often per week do you drink sugar-containing beverages*, *including fruit juice/ alcohol-containing beverages*?’. We dichotomized these variables at the median consumption per week: sugar-containing beverages intake >1 glass per week, alcohol intake ≥4 glasses per week.

#### Health factors

BMI was calculated as self-reported body weight (kilograms) divided by the square root of self-reported height (metres). According to the WHO guidelines overweight was defined as a BMI ≥ 25 kg/m^2^ and obesity as BMI ≥ 30 kg/m^2^ [16].

Self-rated health was measured using a Visual Analogue Scale, consisting of a continuous line ranging from 0 (worst health) to 100 (best health). Participants were asked to indicate how they rated their general health by placing a mark on the line. The Visual Analogue Scale has proven to be a valid, reliable and feasible method of obtaining information on self-rated health [17;18]. Self-rated health was recoded into tertiles. We also asked participants to indicate if they currently suffered from an illness, handicap or other impairment (yes/no).

#### Work factors

Participants reported their current employment status and those employed were asked about the type of work they did. We combined these two questions into one item: i.e. sitting occupation, standing occupation, (heavy) manual work, retired, and other (in education, homemaker, unemployed).

#### Psychological factors

Happiness was measured by a single item rated on a 5-point scale asking *‘How happy are you in general*?’. Answering scale ranged from very happy to very unhappy. We dichotomized the question into unhappy/ neutral and happy.

#### Physical home and neighbourhood environment

Participants reported if they owned a car, and how many screens they had in their household (including desktop computers, laptops, TVs and tablets). We dichotomized the screen variable at the median of >4 screens in the house.

Data on residential density were obtained from the Urban Atlas database (European Environment Agency, 2002) using two categories: high and low residential density (>2/3 and <1/3 of areas covered by residential buildings, respectively). Neighbourhoods were classified as low or high SES on the basis of recent data on neighbourhood median income (i.e. the first and third tertiles, respectively) retrieved from each country’s national statistics office [8]. Residential neighbourhood type was classified for SES and residential density, both dichotomised into low and high.

#### Social neighbourhood environment

Aspects of neighbourhood social capital were measured as previously proposed by Beenackers et al. using a 13-item scale [19]. Items captured interactions and relationships in the neighbourhood such as ‘*the people in my neighbourhood get along with each other well*’. Responses ranged from 1 (totally disagree) to 5 (totally agree). Factor analysis was performed and reliabilities of the three identified constructs were α = 0.83 for ‘social network’ (4 items), α = 0.79 for ‘social cohesion’ (5 items) and α = 0.58 (3 items) for ‘place attachment/sense of belonging’. Based on the Cronbach’s alpha, only social cohesion and social network were considered to be reliable social capital factors. Summary scores of social cohesion and social network were calculated for each individual, with values ranging between 5–25 and 4–20, respectively. Detailed methodology of the factor analysis can be found elsewhere [20].

### Statistical analyses

We excluded participants who did not provide both questionnaire data and valid accelerometer data. Descriptive statistics were used to summarise participant characteristics.

The first model examined univariate associations between the individual and environmental variables and sedentary time among adults (9h or less/more than 9 hours per day) using logistic regression. Given the hierarchical structure of the data (individuals within neighbourhoods), we adjusted the associations for the neighbourhood identifier. Due to the variation in accelerometer wear time, we adjusted all analyses for wear time.

In the second model, all univariate factors from the first step surpassing the statistical threshold of p<0.10 were added simultaneously to a multivariable logistic regression model, adjusted for neighbourhood identifier.

Item-nonresponse ranged from 1% (age) to 31% (self-rated general health). Assuming that data were missing at random, missing values for all variables were imputed using predictive mean matching. All variables described in the methods section were used as predictors in the imputation model to create 30 imputed datasets. Sensitivity analyses were carried out using the imputed dataset and using a cut-off of 10h sedentary time or more per day. All analyses were conducted in SPSS version 22.0.

## Results

Table 1 presents the participant characteristics. In total, 223 adults from the Randstad region (Netherlands) and 133 adults from the Ghent region (Belgium) were included for analyses. Mean minutes spent sedentary per day was 543.8 (SD 88.4) and 54% of the participants were sedentary for at least 9 hours per day. Mean accelerometer wear time was 873.0 (SD 69.8) minutes per day and mean number of valid days was 6.8 (SD 0.6).

**Table 1:** Study sample description.

Characteristics	All participants Mean (SD) or %	Dutch participants Mean (SD) or %	Belgian participants Mean (SD) or %
N	356	223	133
Gender (women)	52%	56%	45%
Age (years)	55.8 (15.6)	57.6 (15.3)	52.8 (15.8)
BMI (kg/m^2^)	24.6 (4.0)	24.6 (4.0)	24.7 (4.1)
Highest education level			
Low	21%	16%	30%
Medium	29%	25%	37%
High	50%	59%	34%
Accelerometer			
Light PA (min/day)	299.1 (76.6)	295.3 (76.9)	305.5 (76.0)
MVPA (min/day)	30.0 (22.6)	28.9 (21.5)	32.0 (24.2)
Sedentary time (min/day)	543.8 (88.4)	557.0 (81.5)	521.6 (95.0)
% Sedentary >9 h/day	54%	61%	43%
Wear time (min/day)	873.0 (69.8)	881.2 (64.6)	859.2 (76.1)
Valid wear days	6.8 (0.6)	6.8 (0.4)	6.6 (0.7)

Between March and August 2014, Belgian (n = 133) and Dutch (n = 223) adults participated | N = number of participants | SD = standard deviation | BMI = body mass index | PA = physical activity | MVPA = moderate to vigorous physical activity

Compared to the full SPOTLIGHT sample in Belgium and the Netherlands, the Belgian sample included in this study consisted of slightly less women (53% vs. 45% respectively, but were comparable with regard to age (53.0, SD 16.7 vs. 52.8yrs, SD 15.8) and BMI (25.4, SD 4.5 vs. 24.7, SD 4.1). The Dutch sample included in this study consisted of slightly more women (54% in full SPOTLIGHT sample vs. 56% in this study), They were also slightly older (54.9, SD 15.9 vs. 57.6yrs, SD 15.3) and had a lower BMI (25.0, SD 3.9 vs. 24.6, SD 4.0 in this study) [8].

Results of the univariate model, as well as results of the multivariable adjusted logistic regression model are presented in Table 2. Results were reasonably similar in univariate and multivariable models. Only multivariable results are described below.

**Table 2:** Univariate and multivariable logistic regression model.

							Univarate model			Multivariable model		
							Adjusted for NBH_NR and wear time			Adjusted for NBH_NR and wear time (N = 317)		
	N	% total	Mean sedentary time min/day	SD	N sedentary >9 h/day	within group %	OR for being sedentary >9 h/day	95% CI		OR for being sedentary >9 h/day	95% CI	
**Overall**	356	100	544	88	193	54						
**SOCIODEMOGRAPHIC FACTORS**												
**Country of residence**												
Netherlands (ref)	223	63	557	81	136	61	1.00			1.00		
Belgium	133	37	522	95	57	43	0.49	0.22	1.13	**0.22**	**0.08**	**0.65**
**Age**												
<35 years (ref)	36	10	554	54	24	67	1.00			1.00		
35–49 years	83	24	517	86	35	42	**0.33**	**0.14**	**0.77**	**0.18**	**0.06**	**0.53**
50–64 years	116	33	558	98	67	58	0.50	0.22	1.14	**0.31**	**0.11**	**0.87**
65+ years	118	33	544	86	66	56	0.66	0.29	1.49	**0.24**	**0.07**	**0.89**
**Gender**												
Man (ref)	167	48	563	99	105	63	1.00					
Women	181	52	527	73	85	47	**0.51**	**0.32**	**0.81**	**0.44**	**0.25**	**0.78**
**Educational level**												
Low (ref)	74	21	532	115	35	47	1.00					
Medium	101	29	542	79	52	51	0.96	0.50	1.83			
High	173	50	553	80	104	60	1.23	0.67	2.24			
**LIFESTYLE FACTORS**												
**MVPA**												
Less than 150 min/week (ref)	154	43	557	98	93	60	1.00			1.00		
150 min/week or more	202	57	534	79	100	50	**0.45**	**0.28**	**0.72**	**0.39**	**0.22**	**0.71**
**Sleep**												
Sleeping for <7 or >9 h/d (ref)	98	28	551	97	55	56	1.00					
Sleeping 7–9 h/day	250	72	540	78	135	54	1.07	0.64	1.78			
**Smoking**												
No (ref)	198	57	537	77	107	54	1.00					
No, but former smoker	115	33	547	90	62	54	1.02	0.62	1.67			
Yes	33	10	588	131	22	67	1.46	0.63	3.39			
**SCB**												
1 time per week or less (ref)	147	50	537	85	77	52	1.00					
More than 1 time per week	147	50	551	90	85	58	1.39	0.85	2.28			
**Alcohol intake**												
Less than 4 glasses per week (ref)	169	50	544	92	88	52	1.00					
4 glasses per week or more	170	50	546	85	98	58	1.19	0.75	1.87			
**HEALTH**												
**Self-reported BMI**												
Normal weight (ref)	208	60	540	83	106	51	1.00			1.00		
Overweight	106	31	551	103	64	60	**1.78**	**1.06**	**2.99**	1.71	0.92	3.19
Obese	31	9	552	75	19	61	2.00	0.86	4.63	2.17	0.81	5.80
**Illness/Handicap/Impairment**												
No (ref)	275	79	544	81	151	55	1.00					
Yes	72	21	545	114	38	53	1.36	0.75	2.46			
**Self-rated general health**												
Low (ref)	90	33	544	81	49	54	1.00					
Medium	91	34	539	84	49	54	0.80	0.43	1.49			
High	91	34	537	88	47	52	0.73	0.39	1.36			
**WORK FACTORS**												
**Employment and type of work**												
Sitting occupation (ref)	115	33	562	75	75	65	1.00			1.00		
Standing occupation	28	8	553	91	16	57	0.55	0.22	1.37	0.52	0.19	1.43
(Heavy) manual work	36	10	504	93	13	36	**0.20**	**0.09**	**0.46**	**0.14**	**0.05**	**0.40**
Retired	123	35	547	99	67	55	0.78	0.44	1.36	0.56	0.21	1.48
Other (in education, homemaker, unemployed)	45	13	517	69	17	38	**0.33**	**0.15**	**0.71**	**0.25**	**0.10**	**0.60**
**PSYCHOLOGICAL FACTORS**												
**Happiness**												
Unhappy/neutral (ref)	56	16	556	111	30	54	1.00					
Happy	293	84	542	84	161	55	0.96	0.52	1.78			
**PHYSICAL HOME AND NEIGHBOURHOOD ENVIRONMENT**												
**Own at least one car**												
No (ref)	39	11	545	92	20	51						
Yes	307	89	543	83	170	55	1.34	0.65	2.74			
**Number of screens in the household (desktop computers, laptops, TVs, tablets)**												
4 or less (ref)	221	63	536	92	113	51						
More than 4	129	37	559	80	78	61	1.31	0.82	2.09			
**Neighbourhood SES**												
Low SES (ref)	175	49	558	94	103	59						
High SES	181	51	530	80	90	50	0.74	0.48	1.16			
**Neighbourhood residential density**												
Low residential density (ref)	191	54	542	90	98	51						
High residential density	165	46	546	86	95	58	1.29	0.83	2.02			
**SOCIAL NEIGHBOURHOOD ENVIRONMENT**			**Mean**	**SD**								
Social cohesion (range = 4–20)			18.3	11.4			1.01	0.94	1.08			
Social network (range = 5–25)			11.4	3.5			**0.93**	**0.87**	**0.99**	**0.92**	**0.85**	**1.00**

Between March and October 2014, Belgian (n = 133) and Dutch (n = 223) adults participated | N = number of participants | OR = odds ratio | CI = confidence interval | NBH_NR = neighbourhood number | ref = reference category | MVPA = moderate to vigorous physical activity | SCB = sugar-containing beverage | BMI = body mass index | SES = socio economic status | bold = statistically significant, p<0.05

Belgian participants had significantly lower odds (OR = 0.22; CI 0.08–0.65) of being sedentary for 9 hours or more per day than those living in the Netherlands. Being aged 35–49 years, 50–64 years or 65 years and older was associated with lower odds of high sedentary time compared to their younger counterparts of 35 years and younger, respectively OR = 0.18 (CI 0.06–0.53), OR = 0.31 (CI 0.11–0.87), and OR = 0.24 (CI 0.07–0.89). Compared to males, women were less likely to sit for 9 hours per day or more (OR = 0.44; CI 0.25–0.78). Those meeting WHO recommendations for being physically active (i.e. 150 minutes or more per week) were less likely to sit more (OR = 0.39; CI 0.22; 0.71) compared to those not meeting the guidelines. Further, those doing (heavy) manual work and those in the ‘other’ group of employment status (being in education, homemakers and unemployed participants) showed lower odds of sitting more than 9 hours a day compared to those having a sitting job, respectively OR = 0.14 (CI 0.05–0.40) and OR = 0.25 (CI 0.10–0.60). Lastly, those with a higher self-reported social network had slightly lower odds for sedentary time (OR = 0.92; CI 0.85–1.00). A sensitivity analysis performed on the imputed dataset revealed comparable results (see S1 Table), except that age (65 years or older) was no longer a significant correlate of sedentary time in the multivariable model. The sensitivity analysis performed with a 10 hour threshold revealed that country of residence and age were no longer a significant correlate of sedentary time in the multivariable model and those having a standing occupation, being retired and high SES were less likely to sit 10 hours or more per day (see S1 Table).

## Discussion

For better understanding of correlates of sedentary time in adults, this study assessed cross-sectional associations between individual and environmental factors and objectively measured sedentary time in Belgian and Dutch adults. Dutch participants were more likely to sit at least 9 hours per day compared to Belgian participants. This is in line with the study of Loyen et al. who used self-report Eurobarometer data of 26,617 respondents [21].

Those aged 35–49, 50–64 or >65 showed lower odds for large amounts of sitting compared to people younger than 35 years. Whilst Rhodes et al. [4] concluded that age was positively associated with self-reported TV viewing, they reported mixed results for general sitting. In contrast to our findings, the updated review of O’Donogue et al. supported a positive association of age and sitting, e.g. the older, the more sitting time [5]. However, most of those studies (18 of the 20 studies) relied on self-reported measures of sedentary time. As people tend to find it difficult to estimate their actual sitting time [10;11], objective sedentary time data were used in the present study showing a negative relationship: the older, the less sitting time. This is in line with the recent published paper by Jones et al., also using objective sedentary behaviour in a large US sample [22]. More studies with objective measures are needed to confirm the direction of this association.

Consistent with some previous literature [5;21;22], our multivariable analyses showed that women were less likely to sit for 9 hours or more per day compared to males. This is also in line the study of Bernaards et al. using data of a large cross-sectional Dutch survey [23]. It might be that women traditionally spent on average more time housekeeping or taking care of the children, which mostly consists of light intensity activities, resulting in less sitting time. Previous studies using objective sedentary time measures show a mixed pattern: Wilson et al. [24] also found that the female gender was inversely associated with sedentariness, whilst both Conroy et al. [25] and Ekelund et al. [26] reported no association, suggesting no clear cut association.

Regarding lifestyle factors, 50% of our participants were meeting the PA recommendations, but also sat still for 9 hours or more per day. There may be an adverse metabolic and health effects from prolonged sitting even if PA recommendations are being met [27]. A recently published meta-analyses of data from more than 1 million men and women suggests that substantial amounts of PA per day seems to attenuate the increased risk of death associated with high sitting time [2]. This further stresses the need to focus on development and implementation of interventions that target both PA and sedentary behaviours [28].

We did not find significant associations of BMI, illness/handicap/impairment and general health with sedentary time. The literature suggests a tendency towards a positive association between a higher BMI and more sitting [5]. Although the univariate model showed a significant correlation, we only found a hint of a dose-response relation between sedentary time and BMI in the multivariable model in our study, but this was not significant. It must be noted that BMI was self-reported, which is known be prone to measurement bias [29], resulting in possible misclassification of BMI.

Regarding work factors, previous literature suggests an association between retirement and leisure screen and sitting time [5]. Yet, this was not reflected in our data. As we could not measure domains of sitting with accelerometry, this limits exploration of associations of retirement and leisure screen and sedentary time in our study population. It may be that they compensate longer duration of screen time with less sitting time during other activities.

Interestingly, our study did not show a significant difference in sedentary time between those with a sitting and standing job. A previous validation and responsiveness study showed that the hip-mounted ActiGraph accelerometer used in the current study struggles to detect the difference between standing still and sitting down [30]. This might clarify why we found no difference between people with a sitting or standing job. Using an inclinometer-based measurement instrument would allow to pick up changes between these postures and therefore better explore these associations.

As expected, the large majority of the individuals with a sitting job was more than 9 h/d sedentary. This may be due to long sedentary working hours or may also be partly explained by evidence suggesting that those with a sitting job often also engage in more sedentary leisure activities [31]. As mentioned above, it may also be that the accelerometer does not distinguish being sedentary and standing time and therefore overestimates time spent sedentary at work. Nevertheless, it might be that people with a sitting occupation struggle to compensate the large amounts of sitting time they accumulate during working outside working hours. This highlights the importance to address the work place as an intervention setting for breaking up prolonged sitting time at work.

In contrast to previous studies, such as Stamatakis et al. [32], we did not find significant socioeconomic differences in level of sedentary time (after adjustment for neighbourhood clusters). As previous studies often reported socioeconomic differences in specific domains of sedentary behaviour [33;34], it may be that these associations cancel each other out when examining total sedentary time objectively.

Although we found a significant correlation between self-reported social network and sedentary time, we did not find any associations between physical and other social environmental characteristics and sedentary time. It may be that sedentary time is more dependent on the built indoor (home) environment than the neighbourhood environment.

### Strengths and limitations

Among the study’s limitations is the cross-sectional study design. Consequently, we could not explore causality of factors associated with sedentary time. The fact that we used a self-reported questionnaire to measure most correlates is another limitation of the study. Where possible, valid and reliable measures were used in the questionnaire, but self-reported measures are susceptible to recall bias and social desirable answers and could have led to measurement bias, such as potential BMI misclassification in our study. In addition, the study sample comprised a small, relatively older, urban population. This may have influenced our results and limits the generalisability of our results to the whole Dutch, Belgian and other populations.

The SPOTLIGHT study’s main aim was to explore determinants of obesity [7]. As we conducted secondary data analyses, we only included variables that had a theoretical basis for possible associations with sedentary behaviour. Future studies should be designed to specifically investigate environmental factors of sedentary behaviour.

Taking these limitations into account, the major strength of this study is the use of objective sedentary time measured with an accelerometer, providing an accurate estimate of actual time spent sedentary compared to self-reported sedentary time measures such as TV viewing, computer use and leisure sitting time. Most participants were compliant with the measurement procedures, with an average of 873 minutes wear time, whilst a minimum of 600 minutes was set. However, as participants were asked to remove the accelerometer during sleep and water-based activities, it is possible sedentary time has been lost through non-wear in those with shorter wear periods, i.e. at the beginning and end of the day when people are tended to be more sedentary.

Yet, as mentioned before, the hip-worn accelerometer does not detect the difference between standing still and sitting down [30], so information about domain-specific sitting behaviour in addition to objective sedentary time could provide supplemented insight into sedentary lifestyles.

## Conclusions

Our findings add to the growing evidence of factors associated to sedentary time among adults. Country of residence, age, gender, meeting PA recommendations, employment status and social network were associated with objective measured sedentary time. These findings may be used to inform the development of strategies and interventions aimed at reducing sedentary time, and to identify those target groups most in need of such efforts. Yet, additional longitudinal research with objective measured sedentary time in combination with self-reported domain-specific sedentary behaviour is needed to get insight into the causality of these cross-sectional findings.

## Supporting information

S1 Questionnaire (DOCX)

S2 Questionnaire (DOCX)

S3 Questionnaire (DOC)

S1 Table Between March and October 2014, Belgian (n = 133) and Dutch (n = 223) adults participated | OR = odds ratio | CI = confidence interval | NBH_NR = neighbourhood number | ref = reference category | MVPA = moderate to vigorous physical activity | SCB = sugar-containing beverage | BMI = body mass index | SES = socio economic status | bold = statistically significant, p<0.05. (DOCX)
