# Supplementary material for: Individual and environmental correlates of objectively measured sedentary time in Dutch and Belgian adults
Source: PLoS One. 2017 Oct 17;12(10):e0186538. doi: 10.1371/journal.pone.0186538 (PMC5645140; doi:10.1371/journal.pone.0186538)
Supplement: S1 Questionnaire — (DOCX) [file pone.0186538.s001.docx]

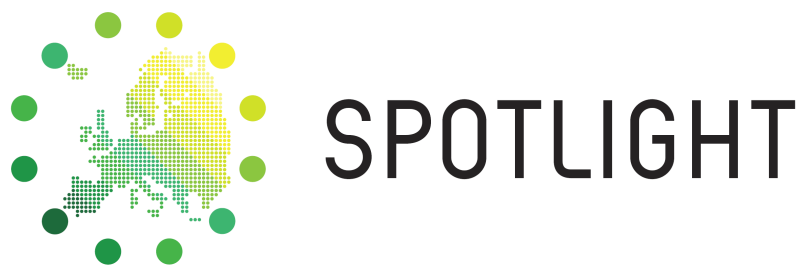


**S1 Appendix - Copy of the online survey questions - English**

**X.** What is your gender?

male Ο

female Ο

**X.** What is your year of birth? 19…….(dropdown menu 1900-1995)

**X.** What is your current main employment status?

currently employed Ο

currently not employed* Ο

retired* Ο

in education Ο

homemaker* Ο

**X.** Does your household own at least one car?

Ο yes

Ο no

**X.** How many electronic screens are there in your household? By screens we mean desktop computers, laptops, televisions, computer tablets, etc.

....... screens (dropdown menu with numbers 0-14, then 15 or more)

**x.** To what extent do you agree with the following statements?

Strongly disagree/disagree/neither agree nor disagree/agree/strongly agree

a. People in this neighbourhood hardly know each other *Ο Ο Ο Ο Ο*

b. I often visit my neighbours in their homes *Ο Ο Ο Ο Ο*

c. I often feel alone in this neighbourhood *Ο Ο Ο Ο Ο*

d. My neighbours visit me on my birthday *Ο Ο Ο Ο Ο*

e. People in this neighbourhood have similar attitudes and values *Ο Ο Ο Ο Ο*

f. I feel at home in this neighbourhood *Ο Ο Ο Ο Ο*

g. If I get the chance, I will move away from this neighbourhood *Ο Ο Ο Ο Ο*

h. Most people in this neighbourhood can be trusted *Ο Ο Ο Ο Ο*

i. Most people in this neighourhood get on with one another *Ο Ο Ο Ο Ο*

j. People in this neighbourhood are willing to help each other *Ο Ο Ο Ο Ο*

k. I borrow or lend things from/to my neighbours *Ο Ο Ο Ο Ο*

l. I can always ask my neighbours if I need advice *Ο Ο Ο Ο Ο*

m. My neighbours would help in case of emergency *Ο Ο Ο Ο Ο*

**X.** What type and amount of physical activity is involved in your work or study?

- Sitting occupation *(you spend most of your time sitting (such as in an office))*
- Standing occupation (*you spend most of your time standing or walking. However, your work does* *not require intense physical effort (e.g. shop assistant, hairdresser, guard, etc.)*
- Manual work (this involves some physical effort including handling of heavy objects and use of tools (*e.g. plumber, electrician, carpenter, etc.)*
- Heavy manual work (this implies very vigorous physical activity including handling of very heavy objects *(e.g farm labourer, bricklayer, construction worker, etc.)*

**X**. How many glasses of sugary drinks do you drink **per week**, including fruit juice?

Ο *One glass per week or less*

Ο *2 glasses per week*

Ο *3 glasses per week*

Ο *4 glasses per week*

Ο *5 glasses per week*

Ο *6 glasses per week*

Ο *7 glasses per week (each day)*

Ο *two glasses per day*

Ο *more than two glasses per day*

**X**. How many glasses of alcohol do you drink **per week**?

Ο *One glass per week or less*

Ο *2 glasses per week*

Ο *3 glasses per week*

Ο *4 glasses per week*

Ο *5 glasses per week*

Ο *6 glasses per week*

Ο *7 glasses per week (each day)*

Ο *two glasses per day*

Ο *more than two glasses per day*

**X.** In general, how happy are you?

1. Very happy Ο

2. Moderately happy Ο

3. No feelings either way Ο

4. Moderately unhappy Ο

5. Very unhappy Ο

**X.** Do you have any longstanding illness, disability or infirmity which limits your daily activities or the work you can do? **Yes** Ο

**No** Ο

**X.** Please place a mark on the line to indicate how you would rate your own health.

(add range under the line: very unhealthy to very healthy)

***************************************************** VAS *******************************************************

**X.** How tall are you (without shoes)? ….centimeters/inches/feet/meters

**X.** How much do you weigh (without shoes/clothes)? …. kilos/pounds/stone

**X.** Do you smoke ?

❑ yes

❑ no, but I have previously been a regular smoker

❑ no, and I have never been a regular smoker

**X.** How many hours do you sleep during an average night? (dropdown menu: 0-16) hours

**X.** What is the highest level of education you have completed?

No formal education Ο

Primary school Ο

Secondary school / high school Ο

Technical qualification Ο

Undergraduate degree Ο

Masters degree or higher Ο
