# Supplementary material for: Individual and environmental correlates of objectively measured sedentary time in Dutch and Belgian adults
Source: PLoS One. 2017 Oct 17;12(10):e0186538. doi: 10.1371/journal.pone.0186538 (PMC5645140; doi:10.1371/journal.pone.0186538)
Supplement: S2 Questionnaire — (DOCX) [file pone.0186538.s002.docx]

**
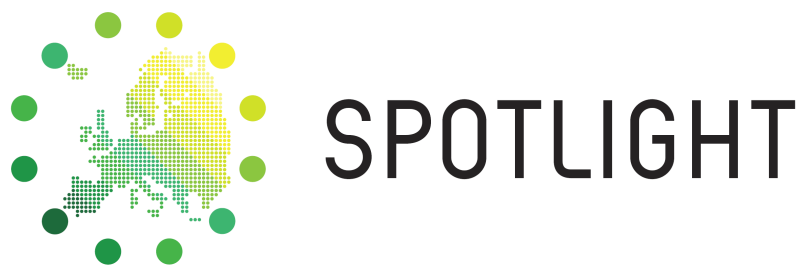
**

**S1 Appendix - Copy of the online survey questions - Netherlands**

**X.** Wat is uw geslacht?

Ο man

Ο vrouw

**X.** In welk jaar bent u geboren?   19……. (1900-1995 keuzemenu)

**X.** Wat is uw arbeidspositie?

ik ben op dit moment werkzaam                                           Ο

ik ben op dit moment niet werkzaam*                                         Ο

ik ben met pensioen / de VUT*                                                      Ο

ik volg een opleiding                                              Ο

ik ben huisman / -vrouw*                                              Ο

**X.** Bezit uw huishouden (tenminste) één auto?

Ο ja

Ο nee

**X.** Hoeveel beeldschermen zijn er in uw huishouden? Met beeldschermen bedoelen we pc’s, laptops, televisies, tablets, enzovoorts.

         ....... beeldschermen (keuzemenu 0,1,2,…14, 15 of meer)

**X.** Hieronder staan stellingen over de mensen in uw buurt. Wilt u bij iedere stelling aangeven in hoeverre u het met de stelling eens of oneens bent?

Helemaal niet mee eens/niet mee eens/neutraal/mee eens/helemaal mee eens

a. De mensen in deze buurt kennen elkaar nauwelijks *Ο Ο Ο Ο Ο*

b. Ik bezoek mijn buren vaak in hun huis

c. Ik voel me vaak alleen in deze buurt

d. Mijn buren komen bij mij op bezoek als ik jarig ben

e. Mensen in deze buurt hebben dezelfde normen en waarden

f. Ik voel me thuis in deze buurt

g. Als ik de kans krijg, verhuis ik uit deze buurt

h. De meeste mensen in deze buurt zijn te vertrouwen

i. De mensen in deze buurt gaan op een prettige manier met elkaar om

j. Mensen in deze buurt zijn bereid om elkaar te helpen
k.Ik leen dingen van mijn buren

l. Als ik advies nodig heb kan ik dat altijd aan mijn buren vragen

m. Mijn buren helpen elkaar in noodgevallen

**X.** Wat voor en hoeveel lichamelijke activiteit verricht u op uw werk?

Ο Zittend beroep (u brengt het grootste deel van uw tijd zittend door (zoals in een kantoor))

Ο Staand beroep (u brengt het grootste deel van uw tijd staand ​​of lopend door. Maar, u hoeft

voor uw werk geen intensieve lichamelijke inspanning te verrichten (bijvoorbeeld winkelbediende, kapper, bewaker, enz.))

Ο Lichamelijk werk (dit vereist een zekere fysieke inspanning, waaronder het hanteren van zware voorwerpen en het gebruik van gereedschap (bijv. loodgieter, elektricien, timmerman, enz.))

Ο Zwaar lichamelijk werk (dit vereist zeer krachtige fysieke activiteit, waaronder het hanteren van zeer zware voorwerpen (bijv. dokwerker arbeider, timmerman, metselaar, bouwvakker, enz.))

**X.** Hoeveel glazen suikerhoudende frisdrank drinkt u **per week,** inclusief vruchtensap?

Ο  *Eén glas per week of minder*

Ο  *2 glazen per week*

Ο *3 glazen per week*

Ο *4 glazen per week*

Ο *5 glazen per week*

Ο *6 glazen per week*

Ο *7 glazen per week (elke dag)*

Ο *twee glazen per dag*

Ο *meer dan twee glazen per dag*

**X.** Hoeveel glazen alcoholhoudende drank drinkt u **per week**?

Ο  *Eén glas per week of minder*

Ο  *2 glazen per week*

Ο *3 glazen per week*

Ο *4 glazen per week*

Ο *5 glazen per week*

Ο *6 glazen per week*

Ο *7 glazen per week (elke dag)*

Ο *twee glazen per dag*

Ο *meer dan twee glazen per dag*

**X.** Hoe gelukkig bent u in het algemeen?

        1. Erg gelukkig                                                 Ο

        2. Een beetje gelukkig                                  Ο

3. Neutraal                               Ο

        4. Een beetje ongelukkig                             Ο

        5. Erg ongelukkig Ο

**X.** Heeft u een langdurige ziekte, handicap of gebrek die uw dagelijkse activiteiten beperkt, of het werk dat u kunt doen? **Ja** Ο

**Nee** Ο

**X.** Plaatst u de markering alstublieft op de lijn hieronder om aan te geven hoe u uw gezondheid zou beoordelen, variërend van de slechtst mogelijke gezondheid (linkerkant) tot de best mogelijke gezondheid (rechterkant).

***************************************************** VAS *******************************************************

**X.** Hoe lang bent u (zonder schoenen)?                           .... centimeter

**X.** Hoeveel weegt u (zonder schoenen of kleren)?          …. kilo

**X.** Rookt u ?

❑ ja

❑ nee, maar ik ben in het verleden een regelmatige roker geweest

❑ nee, en ik ben nooit een regelmatige roker geweest

**X.** Hoeveel uur slaapt u op een gemiddelde nacht? …..uur

**X.** Wat is het hoogste opleidingsniveau dat u heeft afgerond?

geen opleiding afgerond Ο

lagere school/basisonderwijs Ο

lager beroepsonderwijs(bijv. huishoudschool, LEAO, LTS, VMBO) Ο

middelbaar algemeen voortgezet onderwijs (bijv. MAVO, (M)ULO) Ο

middelbaar beroepsonderwijs(bijv. MBO, MTS, MEAO, MHNO, INAS) Ο

hoger algemeen voorbereidend wetenschappelijk onderwijs(bijv. HAVO,

VWO, HBS, MMS, gymnasium, athenaeum) Ο

hoger beroepsonderwijs(bijv. HBO, HTS, HEAO, PABO) Ο

universiteit Ο
