# Supplementary material for: Individual and environmental correlates of objectively measured sedentary time in Dutch and Belgian adults
Source: PLoS One. 2017 Oct 17;12(10):e0186538. doi: 10.1371/journal.pone.0186538 (PMC5645140; doi:10.1371/journal.pone.0186538)
Supplement: S3 Questionnaire — (DOC) [file pone.0186538.s003.doc]

**
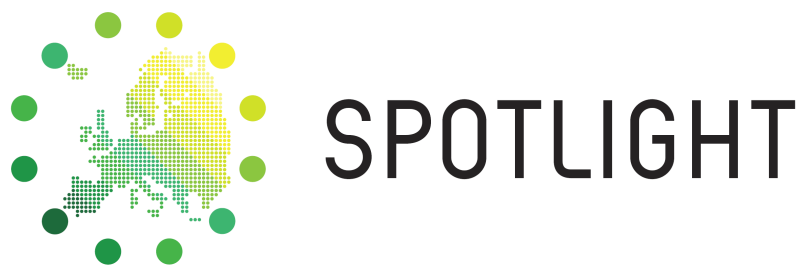
**

**S3 Appendix - Copy of the online survey questions - Belgium**

**X.** Wat is uw geslacht?

Ο man

Ο vrouw

**X.** In welk jaar bent u geboren?                             Drop down menu 1900-1995

**X.** Welke van de onderstaande opties past het best bij u?

ik ben op dit moment werkzaam                                           Ο

ik ben op dit moment niet werkzaam*                                         Ο

ik ben met pensioen*                                                       Ο

ik volg een opleiding                                              Ο

ik ben huisman/-vrouw*                                                     Ο

**X.**  Bezit uw huishouden (tenminste) een auto?

Ο ja

Ο nee

**X.** Hoeveel beeldschermen zijn er in uw huishouden?          ....... beelschermen (keuzemenu: 0,1,2,….13,14 en 15 of meer)

Met beeldschermen bedoelen we pc’s, laptops, televisie’s, computer tablets, enzovoort.

**X.** In welke mate bent u het eens met de volgende stellingen? Geeft u alstublieft één antwoord per stelling.

Helemaal niet mee eens/niet mee eens/neutraal/mee eens/helemaal mee eens

a. Mensen in deze buurt kennen elkaar nauwelijks

b. Ik bezoek mijn buren vaak in hun huis

c. Ik voel me vaak alleen in deze buurt

d. Mijn buren komen bij mij op bezoek als ik jarig ben

e. Mensen in deze buurt hebben dezelfde normen en waarden

f. Ik voel me thuis in deze buurt

g. Als ik de mogelijkheid had, zou ik verhuizen naar een andere buurt

h. De meeste mensen in deze buurt zijn te vertrouwen

i. De mensen in deze buurt gaan op een aangename manier met elkaar om

j. Mensen in deze buurt zijn bereid om elkaar te helpen

k. Ik leen spullen aan of van mijn buren

l. Als ik advies nodig heb over iets, kan ik bij mijn buren terecht

m. Mijn buren helpen me in een noodsituatie

**X.** Wat voor en hoeveel lichamelijke activiteit verricht u op uw werk?

Ο Zittend beroep (u brengt het grootste deel van uw tijd zittend door (zoals in een kantoor))

Ο Staand beroep (u brengt het grootste deel van uw tijd staand ​​of lopend door. Maar, u hoeft

voor uw werk geen intensieve lichamelijke inspanning te verrichten (bijvoorbeeld winkelbediende, kapper, bewaker, enz.))

Ο Lichamelijk werk (dit vereist een zekere fysieke inspanning, waaronder het hanteren van zware voorwerpen en het gebruik van gereedschap (bijv. loodgieter, elektricien, timmerman, etc.)

Ο Zwaar lichamelijk werk (dit vereist zeer krachtige fysieke activiteit, waaronder het hanteren van zeer zware voorwerpen (bijv. dokwerker, arbeider, metselaar, bouwvakker, etc.))

**X.** Hoeveel **glazen frisdrank** drinkt u per week (cola, limonade, fruitsap, etc.)?

Ο  *Eén glas per week of minder*

Ο  *2 glazen per week*

Ο *3 glazen per week*

Ο *4 glazen per week*

Ο *5 glazen per week*

Ο *6 glazen per week*

Ο *7 glazen per week (elke dag)*

Ο *twee glazen per dag*

Ο *meer dan twee glazen per dag*

**X.** Hoeveel **glazen alcoholische drank** drinkt u per week?

Ο  *Eén glas per week of minder*

Ο  *2 glazen per week*

Ο *3 glazen per week*

Ο *4 glazen per week*

Ο *5 glazen per week*

Ο *6 glazen per week*

Ο *7 glazen per week (elke dag)*

Ο *twee glazen per dag*

Ο *meer dan twee glazen per dag*

**X.** Hoe gelukkig bent u in het algemeen?

        1. Erg gelukkig                                                 Ο

        2. Een beetje gelukkig                                  Ο

3. Neutraal                               Ο

        4. Een beetje ongelukkig                             Ο

        5. Erg ongelukkig                         Ο

**X.** Heeft u een langdurige ziekte, handicap of zwakte die uw dagelijkse activiteiten beperkt, of het werk dat u kunt doen? **Ja** Ο

**Nee** Ο

**X.** Plaats de schuifregelaar op de lijn om aan te duiden hoe u uw gezondheid zou beoordelen.

Heel ongezond*************************** VAS **************************Heel gezond

**X.** Hoe groot bent u (zonder schoenen)?                                     …. meter (dropdown menu: 0,1,2) …….. centimeter (dropdown menu: 0-100)

**X.** Hoeveel weegt u (zonder schoenen of kleren)?          …. kilo

**X.** Rookt u ?

❑ ja,

❑ nee, maar ik was vroeger wel een regelmatige roker

❑ nee, en ik ben nooit een regelmatige roker geweest

**44.** Hoeveel uur slaapt u gedurende een gemiddelde nacht? ….. uur (dropdown menu (0-16))

**X**. Wat is het hoogste diploma dat u behaald heeft?

geen opleiding                                                                             Ο

lager onderwijs                                                          Ο

beroepssecundair onderwijs                  Ο

technisch secundair onderwijs                   Ο

algemeen secundair onderwijs (humaniora, kunstonderwijs) Ο

hoger niet-universitair onderwijs                                 Ο

universitair onderwijs          Ο

wil ik niet zeggen         Ο
