# Supplementary material for: Individual and environmental correlates of objectively measured sedentary time in Dutch and Belgian adults
Source: PLoS One. 2017 Oct 17;12(10):e0186538. doi: 10.1371/journal.pone.0186538 (PMC5645140; doi:10.1371/journal.pone.0186538)
Supplement: S1 Table — (DOCX) [file pone.0186538.s004.docx]

**S4 Table 3 - Multivariable logistic regression model of sensitivity analysis for being sedentary > 20 h/day and the imputed dataset**

|  | **Multivariable model sitting >10 h/day** | | | **Multivariable model using imputed data** | | |
| --- | --- | --- | --- | --- | --- | --- |
|  | **Adjusted for NBH_NR and wear time** | | | **Adjusted for NBH_NR and wear time** | | |
|  | **OR for being sedentary >10 h/day** | **95% CI** | | **OR for being sedentary >9 h/day** | **95% CI** | |
| **SOCIODEMOGRAPHIC FACTORS** |  |  |  |  |  |  |
| **Country of residence** |  |  |  |  |  |  |
| Netherlands (ref) |  |  |  | 1.00 |  |  |
| Belgium |  |  |  | **0.34** | **0.13** | **0.90** |
| **Age** |  |  |  |  |  |  |
| <35 years (ref) |  |  |  | 1.00 |  |  |
| 35-49 years |  |  |  | **0.21** | **0.08** | **0.57** |
| 50-64 years |  |  |  | **0.34** | **0.13** | **0.90** |
| 65+ years |  |  |  | 0.40 | 0.11 | 1.43 |
| **Gender** |  |  |  |  |  |  |
| Man (ref) | 1.00 |  |  | 1.00 |  |  |
| Women | **0.26** | **0.13** | **0.52** | **0.50** | **0.29** | **0.85** |
| **Educational level** |  |  |  |  |  |  |
| Low (ref) |  |  |  |  |  |  |
| Medium |  |  |  |  |  |  |
| High |  |  |  |  |  |  |
| **LIFESTYLE FACTORS** |  |  |  |  |  |  |
| **MVPA** |  |  |  |  |  |  |
| Less than 150 min/week (ref) | 1.00 |  |  | 1.00 |  |  |
| 150 min/week or more | **0.26** | **0.13** | **0.55** | **0.41** | **0.24** | **0.71** |
| **Sleep** |  |  |  |  |  |  |
| Sleeping for <7 or >9 h/d (ref) |  |  |  |  |  |  |
| Sleeping 7-9 h/day |  |  |  |  |  |  |
| **Smoking** |  |  |  |  |  |  |
| No (ref) | 1.00 |  |  |  |  |  |
| No, but former smoker | 1.12 | 0.54 | 2.32 |  |  |  |
| Yes | 2.08 | 0.73 | 5.98 |  |  |  |
| **SCB** |  |  |  |  |  |  |
| 1 time per week or less (ref) |  |  |  |  |  |  |
| More than 1 time per week |  |  |  |  |  |  |
| **Alcohol intake** |  |  |  |  |  |  |
| Less than 4 glasses per week (ref) |  |  |  |  |  |  |
| 4 glasses per week or more |  |  |  |  |  |  |
| **HEALTH** |  |  |  |  |  |  |
| **Self-reported BMI** |  |  |  |  |  |  |
| Normal weight (ref) | 1.00 |  |  | 1.00 |  |  |
| Overweight | 1.28 | 0.63 | 2.62 | 1.76 | 0.98 | 3.17 |
| Obese | 0.77 | 0.23 | 2.63 | 1.87 | 0.75 | 4.66 |
| **Illness/Handicap/Impairment** |  |  |  |  |  |  |
| No (ref) | 1.00 |  |  |  |  |  |
| Yes | 1.72 | 0.71 | 4.19 |  |  |  |
| **Self-rated general health** |  |  |  |  |  |  |
| Low (ref) |  |  |  |  |  |  |
| Medium |  |  |  |  |  |  |
| High |  |  |  |  |  |  |
| **WORK FACTORS** |  |  |  |  |  |  |
| **Employment and type of work** |  |  |  |  |  |  |
| Sitting occupation (ref) | 1.00 |  |  | 1.00 |  |  |
| Standing occupation | **0.21** | **0.06** | **0.75** | 0.51 | 0.19 | 1.39 |
| (Heavy) manual work | **0.03** | **0.01** | **0.18** | **0.14** | **0.05** | **0.37** |
| Retired | **0.44** | **0.20** | **0.94** | 0.42 | 0.16 | 1.11 |
| Other (in education, homemaker, unemployed) | **0.29** | **0.09** | **0.96** | **0.23** | **0.10** | **0.55** |
| **PSYCHOLOGICAL FACTORS** |  |  |  |  |  |  |
| **Happiness** |  |  |  |  |  |  |
| Unhappy/neutral (ref) |  |  |  |  |  |  |
| Happy |  |  |  |  |  |  |
| **PHYSICAL HOME AND NEIGHBOURHOOD ENVIRONMENT** |  |  |  |  |  |  |
| **Own at least one car** |  |  |  |  |  |  |
| No (ref) |  |  |  |  |  |  |
| Yes |  |  |  |  |  |  |
| **Number of screens in the household (desktop computers, laptops, TVs, tablets)** |  |  |  |  |  |  |
| 4 or less (ref) |  |  |  |  |  |  |
| More than 4 |  |  |  |  |  |  |
| **Neighbourhood SES** |  |  |  |  |  |  |
| Low SES (ref) | 1.00 |  |  |  |  |  |
| High SES | **0.48** | **0.25** | **0.93** |  |  |  |
| **Neighbourhood residential density** |  |  |  |  |  |  |
| Low residential density (ref) |  |  |  |  |  |  |
| High residential density |  |  |  |  |  |  |
| **SOCIAL NEIGHBOURHOOD ENVIRONMENT** |  |  |  |  |  |  |
| Social cohesion (range = 4-20) |  |  |  |  |  |  |
| Social network (range = 5-25) | **0.92** | **0.83** | **1.01** | **0.92** | **0.85** | **0.99** |

Between March and October 2014, Belgian (n=133) and Dutch (n=223) adults participated | OR = odds ratio | CI = confidence interval | NBH_NR = neighbourhood number | ref = reference category | MVPA = moderate to vigorous physical activity | SCB = sugar-containing beverage | BMI = body mass index | SES = socio economic status | bold = statistically significant, p<0.05
